# Impact of Time-Varying Intensity of Mechanical Ventilation on 28-Day Mortality Depends on Fluid Balance in Patients With Acute Respiratory Distress Syndrome: A Retrospective Cohort Study

**DOI:** 10.3389/fmed.2022.906903

**Published:** 2022-07-28

**Authors:** Weiwei Hu, Suming Zhang, Zhengyu He, Yang Zhou, Ziwen Wang, Yang Zhang, Baohe Zang, Wenjing Zhao, Yali Chao

**Affiliations:** ^1^Department of Critical Care Medicine, The Affiliated Hospital of Xuzhou Medical University, Xuzhou, China; ^2^Department of Critical Care Medicine, School of Medicine, Ren Ji Hospital, Shanghai Jiao Tong University, Shanghai, China

**Keywords:** static driving pressure, mechanical power, fluid balance, 28-day mortality, acute respiratory distress syndrome

## Abstract

**Background:**

Recent studies have mainly focused on the association between baseline intensity of mechanical ventilation (driving pressure or mechanical power) and mortality in acute respiratory distress syndrome (ARDS). It is unclear whether the association between the time-varying intensity of mechanical ventilation and mortality is significant and varies according to the fluid balance trajectories.

**Methods:**

We conducted a secondary analysis based on the NHLBI ARDS Network’s Fluid and Catheter Treatment Trial (FACTT). The primary outcome was 28-day mortality. The group-based trajectory modeling (GBTM) was employed to identify phenotypes based on fluid balance trajectories. Bayesian joint models were used to account for informative censoring due to death during follow-up.

**Results:**

A total of 1,000 patients with ARDS were included in the analysis. Our study identified two phenotypes of ARDS, and compared patients with Early Negative Fluid Balance (Early NFB) and patients with Persistent-Positive Fluid Balance (Persistent-PFB) accompanied by higher tidal volume, higher static driving pressure, higher mechanical power, and lower PaO_2_/FiO_2_, over time during mechanical ventilation. The 28-day mortality was 14.8% in Early NFB and 49.6% in Persistent-PFB (*p* < 0.001). In the Bayesian joint models, the hazard ratio (*HR*) of 28-day death for time-varying static driving pressure [*HR* 1.03 (95% *CI* 1.01–1.05; *p* < 0.001)] and mechanical power [*HR* 1.01 (95% *CI* 1.002–1.02; *p* = 0.01)] was significant in patients with Early NFB, but not in patients with Persistent-PFB.

**Conclusion:**

Time-varying intensity of mechanical ventilation was associated with a 28-day mortality of ARDS in a patient with Early NFB but not in patients with Persistent-PFB.

## Introduction

Acute respiratory distress syndrome (ARDS) is an acute inflammatory lung injury associated with increased vascular permeability, increased lung weight, and loss of aerated lung tissue. The short-term mortality rate ranged from 30 to 50% (1, 2). Lung-protective mechanical ventilation strategies were associated with survival benefits in randomized clinical trials (RCT) involving patients with ARDS (3, 4), and observational studies have recently demonstrated that mitigated driving pressure and mechanical power may increase the survival of ARDS (5–7).

However, previous studies mainly focused on the association between baseline intensity of mechanical ventilation (as measured either by driving pressure or mechanical power) and mortality in ARDS. A retrospective study declared that decreased static driving pressure during the first 24 h of mechanical ventilation was strongly associated with increased survival (7). According to two observational cohorts, a significant effect of high mechanical power during the second 24 h of ventilation on in-hospital mortality was observed in intensive care unit (ICU) patients receiving invasive ventilation for at least 48 h, but not particularly in patients with ARDS (6). Few studies concentrated on the effect of the time-varying intensity of mechanical ventilation on mortality. Whether the strength of the association between the intensity of mechanical ventilation and the mortality of ARDS would remain persistent over time is uncertain.

Fluid balance influenced the dynamic change in the intensity of mechanical ventilation in ARDS. A large RCT evaluated a conservative compared with a liberal fluid strategy in patients with ARDS, and when compared with the liberal strategy, the conservative group had better oxygenation, lung compliance, and a lower plateau pressure during mechanical ventilation within the first 7 days after randomization (8). Put it differently, patients with persistent positive daily fluid balance seem to be characterized by a higher intensity of mechanical ventilation. Whether the dynamic fluid balance could influence the association between the time-varying intensity of mechanical ventilation and mortality remains unknown.

Therefore, we conducted a secondary analysis to elucidate the association between time-varying intensity of mechanical ventilation (as measured either by static driving pressure or mechanical power) and 28-day mortality in patients with ARDS. In addition, we aimed to investigate whether this association would be influenced by the fluid balance trajectories.

## Materials and Methods

### Study Design and Population

We conducted a secondary analysis based on the NHLBI ARDS Network’s Fluid and Catheter Treatment Trial (FACTT) (8). Briefly, the trial included 1,000 patients with ARDS from 2000 to 2005, and all patients were intubated and received positive-pressure ventilation. The detailed description of the inclusion and exclusion criteria is available in the original report. Eligible patients were randomly assigned by a two-by-two factorial design, one arm compared conservative vs. liberal fluid-management, and the other arm compared monitor implemented within a pulmonary artery vs. central venous catheter. The protocols were applied for 7 days. There was no difference in 60-day mortality with either intervention.

All patients enrolled in FACTT were included in the present study. All the data used in present study were approved by the Biologic Specimen and Data Repository Information Coordinating Center (BioLINCC).^1^ Since the study was a secondary analysis based on publicly available database, institutional review board (IRB) approval from our institution was exempted.

### Data Extraction and Outcomes

Demographic data, chronic comorbidities, hemodynamic, respiratory parameters, and laboratory results prior to randomization were recorded. The severity of illness at baseline was determined as measured by the Sequential Organ Failure Assessment (SOFA) score, the Acute Physiology and Chronic Health Evaluation (APACHE) III score, the Charlson Comorbidity index, and the acute lung injury score. Considering that the objective of our study was to explore the interaction between time-varying intensity of mechanical ventilation (which is defined as static driving pressure and mechanical power) and dynamic fluid balance, Day 1 was defined as the day to initiate the protocolized treatment, we collected the fluid balance from Days 1 to 7. Respiratory variables, such as respiratory rate, tidal volume, peak inspiratory pressure, and static driving pressure, were extracted from Day 1 until death, ICU discharge, liberation from mechanical ventilation for more than 48 h or Day 7 in the ICU, whichever occurred first, and the respiratory variables were only recorded on Day 1, 2, 3, 4, and 7 in FACTT. Mechanical power was calculated as 0⋅098 × respiratory rate × tidal volume × [peak inspiratory pressure – (0⋅5 × static driving pressure)].

The primary outcome in the present study was 28-day mortality. Secondary outcomes included 90-day mortality, ventilation-free days in 28 days, ICU-free days in 28 days, and the proportion of dialysis to day 28.

### Statistical Analysis

Values were presented as the proportions for categorical variables and means [standard deviations (SDs)] or medians [interquartile ranges (IQRs)] for continuous variables. Comparisons between groups were made using the *X*^2^-test or Fisher’s exact test for categorical variables and Student’s *t*-test, or the Mann–Whitney *U*-test for continuous variables, as appropriate.

We first attempted to divide patients into groups according to the fluid balance trajectories from Days 1 to 7. Given that not all patients in the liberal-strategy group or the conservative-strategy group complied the protocolized instructions, directly distinguishing the patients based on the different fluid management strategies would be negligent and inappropriately. Hence, group-based trajectory modeling (GBTM) (9) was used to identify phenotypes based on fluid balance trajectories from Days 1 to 7. GBTM is a specialized application of finite mixture modeling and is used to identify groups of individuals following similar trajectories for a particular variable of interest. In the GBTM, variables of fluid balance on each day underwent standardized transformation and log-transformed as appropriate. We estimated models ranging from 2 to 5 classes, and the Akaike information criterion (AIC), Bayesian Information Criteria (BIC), and class size (classes containing relatively small numbers were not considered clinically meaningful) were used to identify the optimal number of phenotypes. GBTM was performed using the traj package in Stata.

The dynamic changes of PaO_2_/FiO_2_ ratio, static compliance, static driving pressure, and mechanical power between phenotypes were compared using a liner mixed-effects model. We included the daily value of static driving pressure or mechanical power as time-varying exposure variables. Considering the non-random dropouts of time-varying variables due to the death during follow-up, we employed Bayesian joint models (10) with shared random effects to examine the effect of a time-varying covariate on a time-to-event outcome, which assumed that all interdependencies between the time-varying exposure and the time-to-event outcome could be explained by latent, subject-specific random effects, after adjustment for baseline covariates. Based on the prior knowledge, baseline variables were purposefully selected into the Bayesian joint model, and included age, BMI, APACHE III Score, PaO_2_/FiO_2_, vasopressor use, catheter type, and fluid management strategy. The primary analysis included all patients with at least one measurement for static driving pressure or mechanical power over time, and secondary analysis were conducted in each clinical phenotypes according to the fluid balance trajectories from Days 1 to 7. We also grouped patients based on fluid management strategy as a sensitivity analysis. Estimation was done using the JMbayes package with JAGS version 4.3.0, using the default settings of the JMbayes package for the JAGS engine (iterations: 900; burn-in: 600; and thinning: 300).

Before the linear mixed-effects model and the Bayesian joint model, we did multiple imputation by chained equations using the MICE package to account for missing data at baseline by generating five imputed datasets for the full study population. The details of missing data are summarized in Supplementary Tables 1, 2. The standardized mean differences (SMDs) and *p*-values were calculated to evaluate the differences between groups or phenotypes, and *p* < 0.05 was considered statistically significant. All statistical analyses were performed using RStudio (version 1.2.5019) and Stata (16.0).

## Results

### Baseline Characteristics

The analysis included data from 1,000 patients with ARDS. The patients had a mean age of 49.7 (16) years, and 534 (53.4%) of them were men. The mean SOFA score was 11.9 (2.6). Static driving pressure was 15.0 (5.4) cmH_2_O and mechanical power was 26.1 (12.1) J/min on Day1. There were 773 survivors and 227 non-survivors, for an overall 28-day mortality rate of 22.7%. The ventilator-free days and ICU-free days during the first 28 days were 20 days (IQR: 14–23) and 18 days (IQR: 11–22), respectively.

### Derivation of Fluid Balance Trajectories of Acute Respiratory Distress Syndrome

**FIGURE 1:**
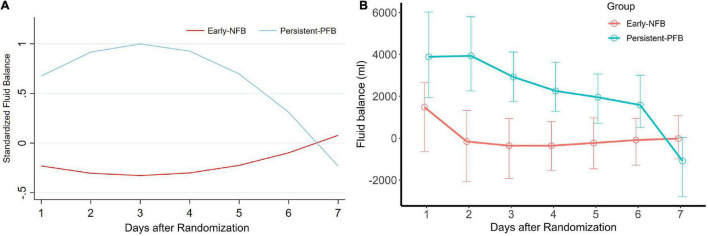
Group-based trajectory model based on the fluid balance trajectories. Using group-based trajectory model, two phenotypes were identified: “Early NFB” and “Persistent-PFB,” Fluid balance were standardized to the mean value of fluid balance **(A)**. Compare to Early NFB, the fluid balance since the day after randomization were significantly higher in Persistent-PFB **(B)**. Early NFB, Early Negative fluid balance; Persistent-PFB, Persistent-Positive fluid balance.

Analysis of the GBTM found that two groups of Acute Respiratory Distress Syndrome (ARDS) patients with distinct fluid balance from Day 1 to Day 7 had the optimal fit (Figure 1). Finally, 772 patients (77.2%) achieved Early Negative fluid balance (Early NFB), and 228 patients (22.8%) had Persistent-Positive fluid balance (Persistent-PFB). The baseline characteristics are shown in Table 1. Patients with Persistent-PFB seems to be characterized by a higher Charlson Comorbidity index, a higher SOFA score, and a higher APACHE III score than those with Early NFB. Compared to patients with Early NFB, patients with Persistent-PFB accompanied by higher tidal volume, higher static driving pressure, higher mechanical power, lower PaO_2_/FiO_2_, and a higher lung injury score. Markers of other organ dysfunction differed significantly between phenotypes (Supplementary Table 3). The 28-day mortality was 14.8% in Early NFB and 49.6% in Persistent-PFB (*p* < 0.001) (Table 2).

**TABLE 1:** Comparisons of baseline characteristics between two phenotypes.

Covariates	All patients	ENFB	PPFB	*P*-value	SMD
N	1,000	772	228	−	−
Age (years)	49.8 (16.0)	49.0 (15.8)	52.3 (16.5)	0.006	0.21
Male, n (%)	534 (53.4)	401 (51.9)	133 (58.3)	0.10	0.13
BMI (Kg/m^2^)	28.6 (7.5)	29.0 (7.3)	27.5 (8.2)	0.012	0.34
Medical ICU, n (%)	663 (66.3)	487 (63.1)	176 (77.2)	<0.001	0.31
Liberal fluid management, n (%)	497 (49.7)	344 (44.6)	153 (67.1)	<0.001	0.466
Primary lung injury, n (%)				<0.001	0.41
Pneumonia	471 (47.1)	355 (46.0)	116 (50.9)		
Sepsis	233 (23.3)	160 (20.7)	73 (32.0)		
Aspiration	149 (14.9)	128 (16.6)	21 (9.2)		
Other	147 (14.7)	129 (16.7)	18 (7.9)		
Charlson comorbidity index	0.0 (0.0–2.0)	0.0 (0.0–2.0)	1.0 (0.0–3.0)	<0.001	0.28
APACHE III score	94.1 (30.9)	89.5 (30.0)	109.6 (28.8)	<0.001	0.68
SOFA score	11.9 (2.6)	11.5 (2.3)	12.3 (2.90)	0.038	0.32
ARDS at baseline, n (%)				0.017	0.22
Mild	217 (21.7)	173 (23.3)	44 (20.3)		
Moderate	496 (49.6)	395 (53.2)	101 (46.5)		
Severe	247 (24.7)	175 (23.6)	72 (33.2)		
Hemodynamic variables					
Heart rate (beats/min)	102.3 (21.1)	100.8 (21.0)	107.1 (20.8)	<0.001	0.30
Systolic blood pressure (mmHg)	113.8 (21.8)	115.6 (21.2)	107.6 (22.7)	<0.001	0.36
Diastolic blood pressure (mmHg)	59.5 (12.7)	60.3 (12.7)	56.7 (12.5)	<0.001	0.29
Mean arterial pressure (mm Hg)	77.2 (14.2)	78.4 (14.0)	73.0 (13.9)	<0.001	0.38
Vasopressor use, n (%)	330 (33)	216 (28.0)	114 (50.0)	<0.001	0.46
Vasopressor dose (μg/min NEE)	8.0 (3.9–15.0)	6.7 (3.2–12.0)	9.0 (5.0–20.0)	0.002	0.46
CVP (mm Hg)	12.1 (5.0)	12.0 (4.9)	12.2 (5.2)	0.75	0.034
PAWP (mmHg)	15.0 (5.22)	14.9 (5.3)	15.4 (5.1)	0.41	0.092
Respiratory variables					
Respiratory rate (breaths/min)	25.5 (8.0)	25.0 (7.9)	27.2 (7.9)	<0.001	0.29
Tidal volume (ml/Kg PBW)	6.69 (1.05)	6.66 (1.03)	6.77 (1.13)	0.19	0.097
Minute ventilation (L/min)	12.3 (4.0)	12.0 (3.8)	13.5 (4.4)	<0.001	0.36
FiO_2_	0.64 (0.21)	0.63 (0.21)	0.69 (0.20)	<0.001	0.27
Plateau pressure (cmH_2_O)	25.0 (21.0–29.0)	25.0 (21.0–29.0)	28.0 (22.0–33.0)	0.10	0.13
PEEP (cmH_2_O)	10.0 (8.0–12.0)	10.0 (8.0–12.0)	10.0 (8.0–14.0)	0.06	0.16
Driving pressure (cmH_2_O)	15.0 (12.0–18.0)	15.0 (12.0–18.0)	15.0 (12.0–19.0)	0.47	0.059
Mechanical power (J/min)	25.9 (13.3)	25.1 (13.1)	28.4 (13.6)	0.003	0.24
Static compliance (ml/cmH_2_O)	28.5 (21.6–37.0)	28.2 (21.3–37.5)	29.4 (22.0–35.3)	0.94	0.04
Ventilatory ratio	1.94 (1.55–2.44)	1.91 (1.54–2.42)	2.01 (1.64–2.49)	0.057	0.14
PaCO_2_ (mmHg)	39.0 (34.0–45.0)	39.0 (34.0–45.0)	38.0 (32.0–44.0)	0.042	0.11
PaO_2_/FiO_2_ (mm Hg)	140.0 (98.3–193)	144.4 (101.7–195.0)	128.9 (87.0–183.3)	0.003	0.17
Lung injury score	2.54 (0.62)	2.49 (0.62)	2.71 (0.58)	<0.001	0.38
Renal variables					
Blood urea nitrogen (mg/dl)	18 (12–30)	16 (11–26)	25 (16–44)	<0.001	0.54
Creatinine (mg/dl)	1.0 (0.7–1.5)	0.9 (0.7–1.4)	1.4 (0.9-1.9)	<0.001	0.46
Urine output (L over previous 24 h)	1.83 (1.07–2.92)	1.96 (1.13–3.08)	1.48 (0.79–2.56)	<0.001	0.22
Hematologic variables					
Hemoglobin (g/dl)	10.4 (1.9)	10.4 (1.9)	10.3 (1.8)	0.21	0.10
Hematocrit (%)	32.5 (6.9)	32.6 (7.0)	32.4 (6.6)	0.70	0.03
Platelets	182.5 (106–261)	186 (112–263)	166 (88–249)	0.028	0.11
White cell count	11.8 (7.2–217.1)	12.0 (7.8–17.0)	10.6 (5.4–17.6)	0.092	0.06
Hepatic variables					
Albumin (g/dl)	2.2 (1.7–2.6)	2.2 (1.8–2.7)	1.9 (1.5–2.3)	<0.001	0.52
Bilirubin (mg/dl)	0.8 (0.5–1.6)	0.8 (0.5–1.5)	0.9 (0.5–2.0)	0.004	0.19
Other variables					
Temperature (°C)	37.5 (1.1)	37.6 (1.0)	37.4 (1.3)	0.11	0.11
Sodium (mmol/L)	138.9 (5.5)	138.8 (5.0)	139.3 (7.0)	0.51	0.05
Chloride (mmol/L)	107.7 (6.9)	107.4 (6.5)	108.6 (8.0)	0.026	0.16
Glucose (mg/dl)	140.2 (70.7)	139.3 (70.5)	143.0 (80.0)	0.51	0.05
Fluid balance (L over previous 24 h)	1.92 (0.44–4.29)	1.77 (0.32–3.84)	2.68 (0.97–5.26)	<0.001	0.36
Glasgow coma scale score	8 (3–11)	8 (4–11)	7 (3–10)	0.024	0.17
pH	7.36 (0.10)	7.37 (0.09)	7.33 (0.11)	<0.001	0.36
Bicarbonate (mmol/L)	21.2 (5.6)	21.8 (5.5)	19.5 (5.7)	<0.001	0.41

*ENFB, early negative fluid balance; PPFB, persistent positive fluid balance; BMI, Body Mass Index; ICU, Intensive Care Unit; APACHE, Acute Physiology and Chronic Health Evaluation; SOFA, Sequential Organ Failure Assessment; NEE, Norepinephrine equivalent; CVP, Central venous pressure; PAWP, pulmonary artery wedge pressure; PEEP, Positive end expiratory pressure; PaO_2_, partial pressure of oxygen; FiO_2_, fraction of inspired oxygen; PaCO_2_, partial pressure of Carbon Dioxide; Ph, Pondus Hydrogenii.*

**TABLE 2:** Treatments and outcomes between two phenotypes.

	ENFB *N* = 772	PPFB *N* = 228	Effect size	*P*-value
**Treatments**				
Conservative fluid management, n (%)	428 (55.4)	75 (32.9)	0.47	<0.001
Pulmonary-artery cather, n (%)	397 (51.4)	116 (50.9)	0.01	0.94
Ever received diuretics*, n (%)	760 (98.4)	225 (98.7)	1.0	0.02
Ever received vasopressors^#^, n (%)	250 (32.4)	168 (73.7)	0.91	<0.001
Ever received RRT, n (%)	48 (6.2)	69 (30.3)	0.66	<0.001
**Outcomes**				
28-day mortality	15.3%	50.0%	0.80	<0.001
90-day mortality	19.7%	57.9%	0.86	<0.001
Ventilation−free days in 28 days	18.3 (7.2)	11.4 (8.2)	0.90	<0.001
ICU-free days in 28 days	17.0 (7.2)	9.8 (7.5)	0.98	<0.001

*ENFB, early negative fluid balance; PPFB, persistent positive fluid balance; ICU, Intensive Care Unit.*

*Ever used diuretics in the study period, included furosemide, chlorothiazide or ethacrynic acid.

*^#^Ever used vasopressor in the study period, included dopamine, norepinephrine, epinephrine, phenylephrine or vasopressin.*

### Changes of Respiratory Variables Over Time and Between Trajectory Groups

**FIGURE 2:**
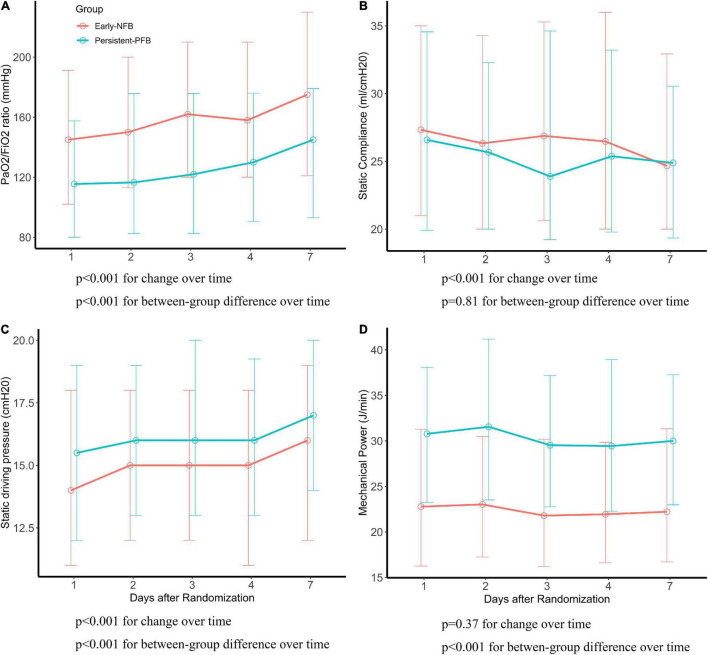
The difference of respiratory parameters between phenotypes. *P*-values for differences with time and for between-group differences using liner mixed effects model. **(A)** PaO_2_/FiO_2_ (mmHg), **(B)** static compliance (ml/cmH_2_O), **(C)** static driving pressure (cmH_2_O), **(D)** mechanical power (J/min).

Respiratory variables between phenotypes during mechanical ventilation within the first 7 days after randomization were presented in Supplementary Table 2. In the linear mixed-effects model, the changes of PaO_2_/FiO_2_ ratio, static compliance, and static driving pressure over time in each phenotype were notably. Over time during mechanical ventilation, the PaO_2_/FiO_2_ ratio and static driving pressure increased, and the static compliance decreased. There was a significant difference between Early NFB and Persistent-PFB with regard to dynamic changes of PaO_2_/FiO_2_ ratio, static driving pressure, and mechanical power, except for static compliance, over time during mechanical ventilation (Figure 2).

### Association Between Intensity of Mechanical Ventilation and 28-Day Mortality

**FIGURE 3:**
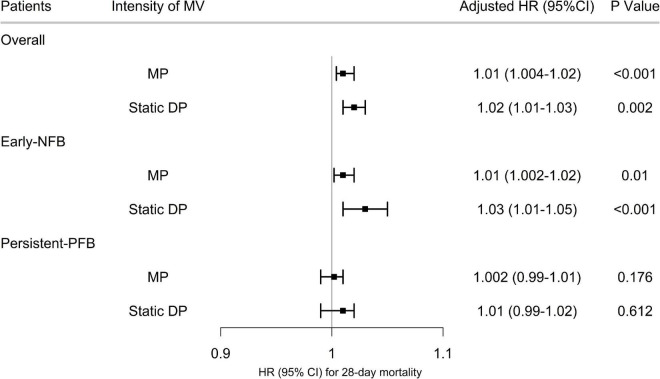
Association between time-varying intensity of mechanical ventilation and 28-day mortality. The hazard ratio and 95% confidence intervals (error bars) in both cohorts were adjusted for the selected covariates. Early NFB, Early Negative fluid balance; Persistent-PFB, Persistent-Positive fluid balance; MV, Mechanical Ventilation; HR, hazard ratio; CI, confidence intervals.

In the Bayesian joint model, after adjusting for age, body mass index (BMI), APACHE III Score, PaO_2_/FiO_2_ ratio, vasopressor use, catheter type, and fluid management strategy, both time-varying static driving pressure [hazard ratio (HR) 1.02 (95% *CI* 1.01–1.03; *p* = 0.002)] and mechanical power [*HR* 1.01 (95% *CI* 1.004–1.02; *p* < 0.001)] were associated with an increased risk of 28-day death in the entire population (Figure 3 and Supplementary Table 4). As for the influence of the dynamic fluid balance, the *HR* of 28-day death for time-varying static driving pressure [*HR* 1.03 (95% *CI* 1.01–1.05; *p* < 0.001)] and mechanical power [*HR* 1.01 (95% *CI* 1.002–1.02; *p* = 0.01)] were significant in patients with Early NFB, but not in patients with Persistent-PFB (Figure 3 and Supplementary Tables 5, 6).

The median duration from intubation to randomization was −2 days (IQR −1 to −1). We conducted a sensitivity analysis in patients who had been intubated 2 days before randomization (93.2%), and the results were similar. We also conducted a sensitivity analysis according to fluid management strategy, the *HR* of 28-day death for time-varying mechanical power was significant in patients with conservative fluid management, while the *HR* of 28-day death for time-varying static driving pressure was significant in patients with liberal fluid management (Supplementary Tables 7, 8).

## Discussion

The major findings of our study can be summarized as follows: (1) time-varying intensity of mechanical ventilation, as measured either by static driving pressure or mechanical power, was associated with increased 28-day mortality in patients with ARDS. (2) We derived two clinical phenotypes of ARDS using trajectory of fluid balance, and detected that such an association was significant in patients with Early NFB. Patients with Early NFB have worse outcomes if they are ventilated with higher driving pressure and/or mechanical power; while patients with persistent-PFB have worse outcome independent of mechanical power and driving pressure because they are already increased, maybe these patients could benefit from early rescue therapies (prone position or ECMO).

Limiting driving pressure and mechanical power have been proposed as targets to reduce mortality in clinical practice of ARDS (11). However, whether we should maintain a lower intensity of mechanical ventilation throughout the entire course of mechanical ventilation has never been questioned. For patients with acute respiratory failure (ARF), exposure to either higher dynamic driving pressure or mechanical power, at any timepoint was associated with higher ICU mortality (12), whereas they adopted dynamic driving pressure instead of static driving pressure, and dynamic driving pressure could be influenced by numerous factors, such as resistive pressures, chest wall compliance, and spontaneous breathing (13, 14). Another observational study identified that lower driving pressure across the ECMO course was associated with better 6-month outcomes in patients with ARDS (15). They used a Cox model with time-dependent covariates. Nevertheless, they ignored the course of mechanical ventilation prior to ECMO, and the Cox model is insufficient to explore such a causal relationship. In our study, after using Bayesian joint models to account for the time-varying variables and the non-random dropouts during follow-up, we detected that both time-varying static driving pressure and mechanical power, at the early phase of mechanical ventilation, were associated with an increased risk of 28-day death for patients with ARDS.

The former mentioned association may be different between groups of ARDS since significant heterogeneity exist. Consequently, identify which patients can benefit more from the interventions targeted at lowering driving pressure or mechanical power was equally important. Unlike previous studies using baseline PaO_2_/FiO_2_ ratio to distinguish the severity of ARDS, we employed trajectories of fluid balance to derive two distinct phenotypes with persistent difference in PaO_2_/FiO_2_ ratio, respiratory mechanical parameters, and disease severity, and concluded that the dynamic intensity of mechanical ventilation had a heterogeneous effect on 28-day mortality according to trajectories of fluid balance. On that basis, this phenomenon could partly explain the insignificant association between driving pressure and mortality in specific groups of patients with ARDS or ARF (16–18).

The main explanations for the insignificant association observed in patients with Persistent-PFB may be as follows. Physiologic variables, such as tidal volume, plateau pressure, peak pressure, respiratory rate, and PEEP, are inter-related in a number of ways since they are both mathematically and physiologically coupled—mathematical coupling occurs because variables are actually derived from each other, physiologic coupling occurs because these variables modify each other when changed in elusive ways—which may result in their complicated association with mortality, with changes to any one variable leading to unpredictable changes in the other dependent variables (19). Secondary, important predictors could have insignificant association ascribed to other measured or unmeasured confounders. For instance, patients in the Persistent-PFB group had a significantly higher age and lower PaO_2_/FiO_2_ on randomization than those in the Early NFB group, and the Persistent-PFB group were more likely to receive vasopressor than those in the Early NFB group, while all these three variables could independently predict the mortality of ARDS in the Persistent-PFB group in our study. Furthermore, although there is a strong physiologic rationale for the importance of driving pressure or mechanical power in ventilated patients with ARDS, it does not mean that an intervention targeted at such a physiologic profile would reduce mortality, especially in patients with ARDS who were severely ill. Randomized trials designed to manipulate or optimize these variables are needed in the future (20). Finally, we only analyzed the association between the intensity of mechanical ventilation within 7 days after randomization and the mortality of ARDS, which may conceal the true effect of driving pressure or mechanical power.

Our study is the first to explore the effect of time-varying intensity of mechanical ventilation on patients with ARDS with different trajectories of fluid balance, and Bayesian joint models were used to adjust for confounders to robust our findings.

Several limitations of the present study should be considered. First, few patients had already been intubated prior to randomization, and we only collected the respiratory variables during the early course—7 days after randomization—but not the entire course of mechanical ventilation due to limited records of FACTT. However, given that 93.2% of patients had been intubated 2 days before randomization, we minimized the impact of mechanical ventilation before randomization according to the sensitivity study mentioned above. Whether the association between the intensity of mechanical ventilation and the outcome of ARDS will remain significant during the entire course of mechanical ventilation needs more studies in future. Second, transpulmonary driving pressure is a more physiologic rationale in ARDS since it represents the pressure actually applied to the lungs and excludes any contribution from the chest wall (14), while it has a lower clinical maneuverability than static driving pressure employed in our study, and previous studies proved that both could independently predict the outcomes of ARDS. Besides, mechanical power was calculated using a simplified formula because of incomplete data. Third, a lack of interventions, such as prone positioning, use of neuromuscular blockers, and extracorporeal membrane oxygenation, might influence the effect of driving pressure or mechanical power on mortality of ARDS. Fourth, since the phenotypes of ARDS in our study were based on the dynamic fluid balance, and the fluid balance is difficult to predict, which might limit the application of fluid balance trajectories in clinical practice. Finally, we clarified a causal relationship between time-varying intensity of ventilation and 28-day mortality of ARDS using Bayesian joint models, which assumed that all baseline variables were measured without error, with no residual confounding and a correctly specified random effect and slope. However, this may be difficult in retrospective study. These effects need to be explored in future studies.

## Conclusion

In conclusion, after using Bayesian joint models, the time-varying intensity of mechanical ventilation during the early course was associated with the increased 28-day mortality of patients with ARDS. Patients with persistent-PFB were characterized by a higher intensity of mechanical ventilation compared to patients with Early NFB, and the association was significant in patient with Early NFB, but not in patients with persistent-PFB. Future studies are need to validate this heterogeneous effect.

## Data Availability Statement

The datasets presented in this study can be found in online repositories. The names of the repository/repositories and accession number(s) can be found below: https://biolincc.nhlbi.nih.gov.

## Ethics Statement

This was a secondary analysis of a prospective RCT which was approved by the institutional review board at each study center, and informed consent was obtained from the patients or their surrogates. All the information was de-identified in the downloaded dataset. Thus, the ethical approval statement and the need for informed consent were waived for this manuscript.

## Author Contributions

WH carried out the design, participated in the collection and assembly of data, and drafted the manuscript. SZ and ZH wrote part of the manuscript. YZO, ZW, YZA, BZ, and WZ participated in the manuscript revision. YC carried out the design, manuscript writing, and final approval of this research. All authors read and approved the final version before submission.

## Conflict of Interest

The authors declare that the research was conducted in the absence of any commercial or financial relationships that could be construed as a potential conflict of interest.

## Publisher’s Note

All claims expressed in this article are solely those of the authors and do not necessarily represent those of their affiliated organizations, or those of the publisher, the editors and the reviewers. Any product that may be evaluated in this article, or claim that may be made by its manufacturer, is not guaranteed or endorsed by the publisher.

## Footnotes

1
https://biolincc.nhlbi.nih.gov

## Abbreviations

ARDS: acute respiratory distress syndrome
RCT: randomized clinical trials
ICU: Intensive Care Unit
ARF: acute respiratory failure
FACTT: Fluid and Catheter Treatment Trial
SOFA: Sequential Organ Failure Assessment
APACHE: Acute Physiology and Chronic Health Evaluation
GBTM: group-based trajectory modeling
AIC: Akaike information criterion
BIC: Bayesian Information Criteria
SMDs: standardized mean differences
Early NFB: Early Negative fluid balance
Persistent-PFB: Persistent-Positive fluid balance
BMI: body mass index.
