# Supplementary material for: Impact of Time-Varying Intensity of Mechanical Ventilation on 28-Day Mortality Depends on Fluid Balance in Patients With Acute Respiratory Distress Syndrome: A Retrospective Cohort Study
Source: Front Med (Lausanne). 2022 Jul 28;9:906903. doi: 10.3389/fmed.2022.906903 (PMC9366012; doi:10.3389/fmed.2022.906903)
Supplement: Supplementary file 1 [file Data_Sheet_1.docx]

Table S1: Percentage of missing data in the variables of interest

|  | **FACTT Cohort** (N=1000) |
| --- | --- |
| Age | 0% |
| Male | 0% |
| BMI (Kg/m^2^) |  |
| Days from ICU admission to randomization (Days) | 0% |
| Ethnicity | 0% |
| Medical ICU | 0% |
| Primary lung injury | 0% |
| Comorbidities |  |
| Hypertension | 0% |
| Diabetes Mellitus | 0% |
| Chronic pulmonary disease | 0% |
| Congestive heart failure | 0% |
| Immune suppression | 0% |
| AIDS | 0% |
| Charlson Comorbidity index | 0.9% |
| APACHE III score | 3.5% |
| SOFA score | 83.6% |
| Hemodynamic variables on Day 1 |  |
| Mean arterial pressure (mm Hg) | 1.7% |
| Vasopressor use | 0% |
| Vasopressor dose (μg/min NEE) | 0% |
| CVP (mm Hg) | 3.7% |
| PAWP (mmHg) | 53.8% |
| Respiratory variables on Day 1 |  |
| Tidal volume (ml/kg PBW) | 9.0% |
| Plateau pressure (cmH2O) | 4.1% |
| PEEP (cmH2O) | 2.3% |
| Driving pressure (cmH2O) | 14.9% |
| Mechanical power (J/min) | 17% |
| PaO_2_/FiO_2_ (mm Hg) | 11.4% |
| Lung injury score | 37.2% |
| FiO_2_ | 5.3% |
| PaO_2_ (mmHg) | 13.3% |
| PaCO_2_ (mmHg) | 13.3% |
| pH | 13.3% |
| Hemoglobin (g/dL) | 2.3% |
| Sodium (mmol/L) | 1.9% |
| Potassium (mmol/L) | 1.8% |
| Glucose (mg/dL) | 2.3% |
| Creatinine (mg/dL) | 2.5% |
| BUN (mg/dL) | 2.8% |
| Chloride (mmol/L) | 2.2% |
| Bicarbonate (mmol/L) | 2.6% |
| Total Protein (g/dL) | 14.7% |
| Albumin (g/dL) | 14.3% |
| 28-day mortality | 0% |
| 90-day mortality | 0% |
| Ventilation‑free days in 28 days | 0% |
| ICU-free days in 28 days | 0% |
| Dialysis to day 28 |  |
| Patients, n (%) | 0% |
| Time to initial dialysis | 0% |

BMI= Body Mass Index；ICU=Intensive Care Unit；AIDS= Acquired Immune Deficiency Syndrome；APACHE= Acute Physiology and Chronic Health Evaluation；SOFA= Sequential Organ Failure Assessment；NEE= Norepinephrine equivalent；CVP= Central venous pressure; PAWP= pulmonary artery wedge pressure; PBW= Predicted body weight; PEEP= Positive end expiratory pressure; PaO_2_=partial pressure of oxygen; FiO_2_=fraction of inspired oxygen; PaCO_2_=partial pressure of Carbon Dioxide; pH=Pondus Hydrogenii; BUN= blood urea Nitrogen.

Table S2: Respiratory values between groups during mechanical ventilation in ICU stay

|  | Day 1 | | Day 2 | | Day3 | | Day4 | | Day7 | |
| --- | --- | --- | --- | --- | --- | --- | --- | --- | --- | --- |
|  | Early-NFB | Persistent-PFB | Early-NFB | Persistent-PFB | Early-NFB | Persistent-PFB | Early-NFB | Persistent-PFB | Early-NFB | Persistent-PFB |
| Patients with MV | 772 | 228 | 710 | 210 | 650 | 189 | 568 | 184 | 393 | 162 |
| PaO_2_/FiO_2_  (mmHg) | 145  (102-191.3) | 115.5  (80-157.5) | 150  (113-200) | 116.5  (82.5-175.8) | 162  (120-210) | 122  (82.5-175.8) | 158  (120-210) | 130  (90.5-176) | 175  (121-230) | 145  (93-179) |
| Missing, n (%) | 100 (12.9) | 14 (6.1) | 109 (15.4) | 15 (7.1) | 79 (12.2) | 13 (6.9) | 95 (16.7) | 19 (10.3) | 72 (18.3) | 24 (14.8) |
| Static Compliance  (ml/cmH20) | 27.3  (21.0-35.0) | 26.6  (19.9-34.6) | 26.3  (20.0-34.3) | 25.7  (20.0-32.3) | 26.9  (20.6-35.3) | 23.9  (19.2-34.6) | 24.7  (20.0-32.3) | 25.4  (19.8-33.2) | 24.7  (20.0-32.9) | 24.9  (19.3-30.5) |
| Missing, n (%) | 131 (17.0) | 32 (14.0) | 185 (26.1) | 40 (19.0) | 207 (31.8) | 32 (16.9) | 204 (35.9) | 37 (20.1) | 163 (41.5) | 49 (30.2) |
| Mechanical Power  (J/min) | 22.8  (16.3-31.3) | 30.8  (23.2-38.1) | 23.0  (17.2-30.5) | 31.6  (23.5-41.2) | 21.8  (16.2-30.2) | 29.5  (22.8-37.2) | 21.9  (16.6-31.3) | 29.4  (22.3-38.9) | 22.2  (16.7-31.3) | 30.0  (23.0-37.3) |
| Missing, n (%) | 136 (17.6) | 34 (14.9) | 184 (25.9) | 37 (17.6) | 212 (32.6) | 35 (18.5) | 211 (37.1) | 38 (20.6) | 166 (42.4) | 50 (30.9) |
| Static driving pressure  (cmH20) | 14 (11-18) | 15.5 (12-19) | 15 (12-18) | 16 (13-19) | 15 (12-18) | 16 (13-20) | 15 (11-18) | 16 (13-19.3) | 16 (12-19) | 17 (14-20) |
| Missing, n (%) | 121 (15.7) | 28 (12.3) | 163 (23.0) | 36 (17.1) | 193 (29.7) | 30 (15.9) | 190 (33.4) | 36 (19.6) | 158 (40.2) | 46 (28.4) |

MV=mechanical ventilation; PaO_2_=partial pressure of oxygen; FiO_2_=fraction of inspired oxygen

Table S3: Additional baseline characteristics between groups stratified by time-varying fluid balance

| Covariates | All patients | Early-NFB | Persistent-PFB | P value |
| --- | --- | --- | --- | --- |
| N | 1000 | 772 | 228 | - |
| Days from ICU admission to  randomization (Days) | 1 (1-2) | 1 (1-2) | 1 (1-2) | 0.16 |
| Days from intubation to  randomization (Days) | 1 (0-2) | 1 (1-2) | 1 (0-1) | 0.029 |
| Ethnicity, n (%) |  |  |  | 0.68 |
| White | 641 (64.1) | 500 (64.8) | 141 (61.8) |  |
| Black | 217 (21.7) | 163 (21.1) | 54 (23.7) |  |
| Other | 142 (14.3) | 109 (14.1) | 33 (14.5) |  |
| Comorbidities, n (%) |  |  |  |  |
| Hypertension | 240 (24.0) | 180 (23.3) | 60 (26.3) | 0.399 |
| Diabetes Mellitus | 173 (17.3) | 119 (15.4) | 54 (23.7) | 0.005 |
| Chronic pulmonary disease | 62 (6.2) | 45 (5.8) | 17 (7.5) | 0.460 |
| Congestive heart failure | 27 (2.7) | 22 (2.8) | 5 (2.2) | 0.760 |
| Immune suppression | 78 (7.8) | 57 (7.4) | 21 (9.2) | 0.445 |
| AIDS | 71 (7.1) | 50 (6.5) | 21 (9.2) | 0.206 |
| Radiological opacity, n (%) |  |  |  | 0.027 |
| 2 quadrant | 62 (6.2) | 44 (5.7) | 18 (7.9) |  |
| 3 quadrant | 159 (15.9) | 135 (17.5) | 24 (10.6) |  |
| 4 quadrant | 776 (77.6) | 591 (76.8) | 185 (81.5) |  |

F AIDS= Acquired Immune Deficiency Syndrome；

Table S4: Association between time-varying intensity of mechanical ventilation and 28-day mortality in overall patients with ARDS

|  | Time-varying mechanical power | | Time-varying driving pressure | |
| --- | --- | --- | --- | --- |
|  | HR (95%CI) | P value | HR (95%CI) | P value |
| **Baseline variables** |  | | | |
| Age | 1.02 (1.01-1.03) | <0.001 | 1.02 (1.01-1.03) | <0.001 |
| BMI | 0.99 (0.97-1.005) | 0.16 | 0.98 (0.97-1.002) | 0.088 |
| APACHE III Score | 1.02 (1.01-1.02) | <0.001 | 1.02 (1.01-1.02) | <0.001 |
| PaO_2_/FiO_2_ | 0.99 (0.99-0.99) | <0.001 | 0.99 (0.99-0.99) | <0.001 |
| Vasopressor use (1^st^ 24 h) | 1.23 (0.92-1.67) | 0.17 | 1.33 (0.99-1.79) | 0.058 |
| Catheter Type |  |  |  |  |
| Use of CVP | Reference | — | Reference | — |
| Use of PAC | 0.96 (0.74-1.27) | 0.75 | 0.99 (0.76-1.30) | 1.00 |
| Fluid management strategy |  |  |  |  |
| Conservative strategy | Reference | — | Reference | — |
| Liberal strategy | 1.10 (0.84-1.45) | 0.47 | 1.12 (0.87-1.47) | 0.40 |
| **Time-varying variables** |  | | | |
| Driving pressure | — | — | 1.02 (1.01-1.03) | 0.002 |
| Mechanical power | 1.01 (1.004-1.02) | <0.001 | — | — |

BMI= Body Mass Index； APACHE= Acute Physiology and Chronic Health Evaluation; PaO_2_=partial pressure of oxygen; FiO_2_=fraction of inspired oxygen; CVP= Central venous pressure; PAC= pulmonary artery catheter.

Table S5: Association between time-varying intensity of mechanical ventilation and 28-day mortality in ARDS patients with Early-NFP.

|  | Time-varying mechanical power | | Time-varying driving pressure | |
| --- | --- | --- | --- | --- |
|  | HR (95%CI) | P value | HR (95%CI) | P value |
| **Baseline variables** |  | | | |
| Age | 1.02 (1.008-1.03) | <0.001 | 1.02 (1.008-1.03) | <0.001 |
| BMI | 0.95 (0.92-0.98) | <0.001 | 0.95 (0.92-0.98) | <0.001 |
| APACHE III Score | 1.03 (1.02-1.03) | <0.001 | 1.03 (1.02-1.03) | <0.001 |
| PaO_2_/FiO_2_ | 0.99 (0.99-0.99) | <0.001 | 0.99 (0.99-0.99) | <0.001 |
| Vasopressor use (1^st^ 24 h) | 0.68 (0.43-1.05) | 0.076 | 0.72 (0.47-0.98) | <0.001 |
| Catheter Type |  |  |  |  |
| Use of CVP | Reference | — | Reference | — |
| Use of PAC | 1.06 (0.72-1.54) | 0.77 | 1.05 (0.70-1.55) | 0.83 |
| Fluid management strategy |  |  |  |  |
| Conservative strategy | Reference | — | Reference | — |
| Liberal strategy | 0.93 (0.64-1.35) | 0.71 | 0.94 (0.64-1.38) | 0.77 |
| **Time-varying variables** |  | | | |
| Driving pressure | — | — | 1.03 (1.01-1.05) | <0.001 |
| Mechanical power | 1.01 (1.002-1.02) | 0.01 | — | — |

BMI= Body Mass Index； APACHE= Acute Physiology and Chronic Health Evaluation; PaO_2_=partial pressure of oxygen; FiO_2_=fraction of inspired oxygen; CVP= Central venous pressure; PAC= pulmonary artery catheter.

Table S6: Association between time-varying intensity of mechanical ventilation and 28-day mortality in ARDS patients with Persistent-PFB.

|  | Time-varying mechanical power | | Time-varying driving pressure | |
| --- | --- | --- | --- | --- |
|  | HR (95%CI) | P value | HR (95%CI) | P value |
| **Baseline variables** |  | | | |
| Age | 1.02 (1.01-1.04) | <0.001 | 1.02 (1.01-1.04) | <0.001 |
| BMI | 1.02 (0.99-1.05) | 0.058 | 1.02 (0.99-1.04) | 0.062 |
| APACHE III Score | 1.006 (0.99-1.01) | 0.07 | 1.006 (0.99-1.01) | 0.068 |
| PaO_2_/FiO_2_ | 0.99 (0.99-0.99) | 0.018 | 0.99 (0.99-0.99) | 0.01 |
| Vasopressor use (1^st^ 24 h) | 1.68 (1.09-2.58) | 0.018 | 1.74 (1.15-2.72) | 0.01 |
| Catheter Type |  |  |  |  |
| Use of CVP | Reference | — | Reference | — |
| Use of PAC | 0.89 (0.60-1.29) | 0.57 | 0.92 (0.62-1.36) | 0.70 |
| Fluid management strategy |  |  |  |  |
| Fluid management (Conservative) | Reference | — | Reference | — |
| Fluid management (Liberal) | 0.89 (0.60-1.37) | 0.56 | 0.92 (0.62-1.36) | 0.69 |
| **Time-varying variables** |  | | | |
| Driving pressure | — | — | 1.009 (0.99-1.022) | 0.18 |
| Mechanical power | 1.002 (0.99-1.01) | 0.61 | — | — |

BMI= Body Mass Index； APACHE= Acute Physiology and Chronic Health Evaluation; PaO_2_=partial pressure of oxygen; FiO_2_=fraction of inspired oxygen; CVP= Central venous pressure; PAC= pulmonary artery catheter.

Table S7: Association between time-varying intensity of mechanical ventilation and 28-day mortality in ARDS patients with conservative fluid management

|  | Time-varying mechanical power | | Time-varying driving pressure | |
| --- | --- | --- | --- | --- |
|  | HR (95%CI) | P value | HR (95%CI) | P value |
| **Baseline variables** |  | | | |
| Age | 1.02 (1.01-1.03) | <0.001 | 1.02 (1.01-1.03) | <0.001 |
| BMI | 0.99 (0.97-1.02) | 0.88 | 0.99 (0.96-1.02) | 0.67 |
| APACHE III Score | 1.02 (1.01-1.03) | <0.001 | 1.02 (1.01-1.03) | <0.001 |
| PaO_2_/FiO_2_ | 0.96 (0.67-1.42) | 0.83 | 0.99 (0.99-0.99) | 0.008 |
| Vasopressor use (1^st^ 24 h) | 0.75 (0.48-1.15) | 0.22 | 0.67 (0.44-1.06) | 0.08 |
| Catheter Type |  |  |  |  |
| Use of CVP | Reference | — | Reference | — |
| Use of PAC | 0.99 (0.99-0.99) | 0.032 | 0.93 （0.63-1.44） | 0.72 |
| **Time-varying variables** |  | | | |
| Driving pressure | — | — | 1.02（0.99-1.04） | 0.11 |
| Mechanical power | 1.01 (1.003-1.02) | 0.004 | — | — |

BMI= Body Mass Index； APACHE= Acute Physiology and Chronic Health Evaluation; PaO_2_=partial pressure of oxygen; FiO_2_=fraction of inspired oxygen; CVP= Central venous pressure; PAC= pulmonary artery catheter.

Table S8: Association between time-varying intensity of mechanical ventilation and 28-day mortality in ARDS patients with liberal fluid management

|  | Time-varying mechanical power | | Time-varying driving pressure | |
| --- | --- | --- | --- | --- |
|  | HR (95%CI) | P value | HR (95%CI) | P value |
| **Baseline variables** |  | | | |
| Age | 1.02 (1.009-1.03) | <0.001 | 1.02 (1.01-.03) | <0.001 |
| BMI | 0.98 (0.95-1.003) | 0.092 | 0.97 (0.95-1.002) | 0.072 |
| APACHE III Score | 1.02 (1.009-1.02) | <0.001 | 1.02 (1.009-1.02) | <0.001 |
| PaO_2_/FiO_2_ | 0.99 (0.99-0.99) | <0.001 | 0.99 (0.99-0.99) | <0.001 |
| Vasopressor use (1^st^ 24 h) | 0.85 (0.58-1.27) | 0.43 | 0.81 (0.54-1.19) | 0.29 |
| Catheter Type |  |  |  |  |
| Use of CVP | Reference | — | Reference | — |
| Use of PAC | 1.02 (0.71-1.45) | 0.95 | 1.08 (0.76-1.57) | 0.69 |
| **Time-varying variables** |  | | | |
| Driving pressure | — | — | 1.02 (1.005-1.03) | 0.006 |
| Mechanical power | 1.007 (0.99-1.02) | 0.15 | — | — |

BMI= Body Mass Index； APACHE= Acute Physiology and Chronic Health Evaluation; PaO_2_=partial pressure of oxygen; FiO_2_=fraction of inspired oxygen; CVP= Central venous pressure; PAC= pulmonary artery catheter.
